# Validating the use of a local developmental dataset for *Cochliomyia macellaria* (Diptera: Calliphoridae) to estimate the time of placement of various carrion types and sizes in Texas, United States, during summer

**DOI:** 10.1093/jme/tjaf167

**Published:** 2025-11-14

**Authors:** Sienna N McPeek, Amber E MacInnis, Jeffery K Tomberlin

**Affiliations:** Department of Entomology, Texas A&M University System, College Station, TX 77845, USA; Department of Entomology, Texas A&M University System, College Station, TX 77845, USA; Department of Entomology, Texas A&M University System, College Station, TX 77845, USA

**Keywords:** forensic entomology, Cochliomyia macellaria, ecological validation, species coexistence, developmental models

## Abstract

Accurate estimation of the time of colonization (TOC) is a cornerstone of forensic entomology, yet direct validation of laboratory-derived development datasets in field contexts remains rare. Within validation studies, when the TOC is unknown, the time of placement (TOP)—the point at which remains become accessible to insects (e.g,. Diptera)—can serve as a proxy. This study evaluated the accuracy of *Cochliomyia macellaria* (Diptera: Calliphoridae) development estimates under varying ecological conditions by calculating the estimated-to-actual TOP ratio (%). Factors examined included carcass type (small, medium, and large mammals; large birds), thermal summation method (accumulated degree days [ADD] vs. accumulated degree hours [ADH]), emergence timing (first vs. last emergence), development dataset tissue source (porcine vs. equine), and species coexistence with *Chrysomya rufifacies* (Diptera: Calliphoridae). A full-factorial, aligned rank-transformed ANOVA was conducted using three replicates per carcass type across two Texas field sites in 2023 and 2024. Using ADD, first-emergence data, and porcine-derived tissue consistently yielded the highest TOP ratios (93.9%, 96.3%, and 93.4%, respectively). Precision was greatest in small mammals and lowest in large mammals. Although ADD-based estimates generally outperformed ADH, ADH occasionally demonstrated greater consistency under certain contexts. Species coexistence and trial year showed context-dependent influences on TOP ratio outcomes. These findings underscore the importance of incorporating ecological and biological variability when applying development studies to forensic casework and highlight the robustness of ADD calculations and early-emergence sampling for estimating TOC in the field.

## Introduction

In forensic entomology, the time of colonization (TOC) refers to when insects first deposit their offspring on a deceased or living vertebrate host (Amendt et al. 2007, Tomberlin et al. 2011, Cammack et al. 2015). Estimating the TOC assists law enforcement with reconstructing timelines for minimum time from death to discovery (minimum postmortem interval (minPMI)) or abuse/neglect (Catts and Goff 1992, Goff 1993, Tomberlin et al. 2011). These estimates are grounded in the predictable development of such insects (Kerkut and Taylor 1958, Denno and Cothran 1976, Dugan 2003, Parmenter and Macmahon 2009); of course, proper species identification, in conjunction with published development data, is essential (Byrd and Allen 2001, Arnaldos et al. 2004, Sharanowski et al. 2008, Shi et al. 2009, Núñez-Vázquez et al. 2013).

Despite the reliance on laboratory-derived growth rate data for estimating the TOC, relatively few studies have validated these data under natural field conditions (VanLaerhoven 2008, Faris et al. 2020). Existing validation studies are region-specific, complicating the broader applicability of these data to regions that differ in abiotic and biotic factors (National Research Council 2009). Additional factors such as carcass type and size (Kneidel 1984), monitoring frequency of insect activity (i.e., hourly vs daily observations) (Moleón et al. 2017), and geographic climate variability (Tomberlin et al. 2011) can have compromising effects on TOC estimates as well. In addition to external variables, biological factors impacting insect behavior and physiology may also introduce error.

Larval density (i.e., formation of dipteran maggot masses, number of fly larvae per gram of substrate) is another factor often overlooked in laboratory settings. In nature, larvae congregate in dense larval masses, generating significant metabolic heat that can decouple developmental rate from ambient temperature (Slone and Gruner 2007). These larval masses can raise localized temperatures by more than 20 °C, potentially reducing development time (Anderson 2019). In contrast, laboratory studies typically use low larval densities to avoid such thermal artifacts (Charabidze et al. 2011), which may lead to underestimations or overestimations when these data are applied in forensic casework. Similarly, differences in development can be population-specific. For example, *Lucilia sericata* (Robineau-Desvoidy) (Diptera: Calliphoridae) from Sacramento, CA, United States, had significantly different growth rates than *L. sericata* from San Diego, CA, United States (Gallagher et al. 2010). Consequently, applying development data derived from one geographic population to another could introduce error in TOC estimates. This response was echoed in a previous study with the secondary screwworm, *Cochliomyia macellaria* Fabricius (Diptera: Calliphoridae) (Owings et al. 2014), which emphasized the variability in growth rates for this species across three ecoregions in TX, United States, and its implications for forensic casework.

Interspecific interactions can also significantly shift blow fly development timelines. *Cochliomyia macellaria* frequently co-occurs with *Chrysomya rufifacies* Macquart (Diptera: Calliphoridae), an invasive competitor and intraguild predator of *Co. macellaria* larvae (Brundage et al. 2014). These interactions may reduce competition through direct consumption but can also trigger non-consumptive effects, such as accelerated growth rates in *Co. macellaria* third instars when exposed to *Ch. rufifacies* excretions/secretions (Flores et al. 2017). Wells and LaMotte (2017) further highlight the ecological impact of predatory interactions in blow fly succession, noting that such interspecific dynamics may alter colonization patterns and larval survivorship. Thus, coexistence with predatory and/or competitive species can introduce variability into developmental timelines and confound TOC estimation.

The 2009 National Academy of Sciences report emphasized the need to assess error rates in forensic disciplines (National Research Council 2009). This report has driven efforts to standardize methods and evaluate the reliability of entomological evidence in a forensic context, such as TOC as related to the minPMI under certain assumptions (Tarone and Sanford 2017). Presently, frameworks exist to systematically study colonization patterns (Amendt et al. 2007, Tomberlin et al. 2011), insect attraction (Ma et al. 2012), and development (Boatright and Tomberlin 2010) under various decomposition scenarios. Although validation studies have been published (Núñez-Vázquez et al. 2013, Faris et al. 2020, Matuszewski 2021), field studies with *whole* vertebrate carrion are rare. Perez et al. (2016) emphasized the need for realistic, field-based validation of laboratory-based studies that typically utilize small samples or artificial substrates (eg, 50 g liver in rearing cups), but this may not reflect natural decomposition dynamics, resulting in the production of inaccurate TOC estimates. Furthermore, studies typically utilize tissues from non-human carcasses, such as swine (VanLaerhoven 2008, Reibe-Pal and Madea 2015, Weatherbee et al. 2017, Pittner et al. 2020) or rodents (Bourel et al. 2003, Tarone and Foran 2008). Predictive accuracy in these studies for estimating insect age of samples varies widely—spanning from a 2 h difference to 4 d or more—emphasizing the need for more studies that incorporate a variety of whole-carcass replicates of varying origin and biomasses.

Validation studies with vertebrate remains in the field can be limited in value for numerous reasons. For example, assessing the accuracy and precision when using the age of insects collected is difficult due to the actual TOC of the remains being unknown. This limitation has been circumvented by studies that utilize animal tissue being inoculated with a controlled number of blow fly eggs, then assessing growth patterns over time under such conditions. Indicators of larval age are also study-specific; for example, larval length and feeding-stage categorization were of appropriate use within a controlled lab study (Tarone and Foran 2008), while instar was more appropriate for estimates from a single species in a controlled field study (Faris et al. 2020). In fact, there are multiple limitations within existing validation studies, including but not limited to: tissue type used, controlled densities, utilization of a single colonizer species rather than the whole fly community, and the subsequent influence on the growth rates of specimens in question.

Given the challenges with knowing the exact TOC of vertebrate remains, an alternative measure can be the time of placement (TOP), or deployment of the remains in the field, as it should be explicitly known (Owings et al. 2022). The primary purpose of this study was to evaluate the estimated-to-actual TOP ratios (%) based on *Co. macellaria* development to the adult stage on vertebrate carcasses of different sizes and types across locations and years when applying a previously published dataset (Boatright and Tomberlin 2010). Specifically, our objectives were to (1) evaluate *Co. macellaria* estimates of TOP across carcass types, year, site, thermal summation method, emergence timing, development study tissue type, and species coexistence when using Boatright and Tomberlin (2010) development data; and (2) determine the contextual limits (e.g., accuracy and precision) of applying laboratory-derived development data to field-derived emergence timing.

## Materials and Methods

### Carcass Types Used

Carcasses were assigned to biomass classes by pre-deployment weight—small (≤1.5 kg), medium (1.6–3.0 kg), and large (3.1–5.5 kg)—with rabbits (*Oryctolagus cuniculus*) and chickens (*Gallus gallus domesticus*) represented in each class. Fully intact and frozen carcasses (euthanized with CO_2_) were sourced from a commercial supplier (RodentPro.com, Inglefield, IN, United States), and no animal use protocol was required, as animals were not euthanized specifically for this research. Smaller vertebrates such as rabbits and poultry have been widely used in decomposition studies when larger cadavers are unavailable (Goff 2009).

The variation in whole-carcass types across trials enabled an evaluation of whether developmental datasets for *Co. macellaria*—originally generated from larvae reared on excised porcine and equine muscle tissues under constant temperature conditions (Boatright and Tomberlin 2010)—can be applied to estimate TOP under field conditions. Boatright and Tomberlin (2010) specifically examined how tissue composition influences developmental rate using excised porcine and equine tissue, with equine tissue included to represent distinct muscle and moisture conditions. Recognizing that tissue composition varies, this validation aims to address a key forensic challenge: assessing whether developmental data can be applied reliably across tissue types and determining the degree to which such datasets accurately and precisely estimate TOP across carcass types and biomasses. While humans would be categorized within a larger-mammalian size class, forensic and wildlife casework often involves remains that are partial, dismembered, or otherwise reduced in biomass. Evaluating the developmental predictability across carcass sizes provides a practical means to determine the robustness of existing datasets when applied to remains of varying biomass or exposure to the environment.

### Carcass Preparation and Field Sites

Frozen carcasses received from the supplier were labeled and stored at −20 °C until needed. Prior to each trial, carcasses were thawed at room temperature for 24 h per previous study methods (Schoenly et al. 2015), then individually weighed in grams using an OHaus Adventurer Pro AV64 balance (Ohaus Corporation, Pine Brook, NJ, United States). Two trials were conducted—one in June to July 2023 and one in June to July 2024—in Central TX, United States: Ecology and Natural Resource Teaching Area (ENRTA) in College Station (30°34′16″N, 96°22′00″W) and Coulter Airfield (C.A.) in Bryan, TX, United States (30°43′09″N, 96°19′57″W) using identical methods across both sites. These sites are both characterized as non-woody grasslands comprised largely of bluestem, *Andropogon gerardi*, and Indian grass, *Sorghastrum nutans*, while surrounding forested areas are scattered with patches of American elm, *Ulmus americana*, juniper, *Juniperus virginiana*, hackberry, *Celtis laevigata*, and water oak, *Quercus nigra*. Although the patchy wooded areas provide some shade to the overall landscape, all carcasses across both sites were placed in full sun as an ecological constant.

### Carcass Deployment

Carcasses were placed at field sites at sunrise—aligned with crepuscular fly activity patterns (Mohr and Tomberlin 2014)—using National Oceanic and Atmospheric Administration (NOAA) sunrise data, and were placed in the field using a random number generator within a predefined site grid. To ensure independence, carcasses were positioned ∼25 m apart (Weidner et al. 2015). Each individual intact whole carcass was placed in a plastic pan (Sterilite, MA, United States; 59.7 × 42.9 × 14.9 cm) containing a 2.5 to 5.0 cm layer of fine playground sand and pea gravel (Kolor Scape, GA, United States), which acted as a pupation substrate and containment barrier. Carcasses were centrally positioned within each pan and covered with galvanized hardware cloth (Garden Craft, mesh 1.3 cm, 91.4 × 152.4 cm; Walmart Inc., AR, United States) to prevent vertebrate scavenger access. Covers were secured using four galvanized rebar stakes with one hammered into the ground on each side (3.51 × 0.64 × 30.48 cm; Vodaland, Amazon, TX, United States).

### Monitoring and Transport to Laboratory

Insect activity was monitored twice daily (07:00–09:00 h and 17:00–19:00 h) during the first 3 days post-deployment, when colonization typically occurs, and once daily thereafter (07:00–09:00 h), consistent with protocols to capture peak activity while minimizing disturbance (Anderson 2000). Based on prior methods (Sawyer et al. 2022), predicted accumulated degree hours were calculated before each trial using NOAA weather data from Easterwood Airport (CLL) in College Station, TX, United States, located approximately 1.0 to 6.0 km from the field sites (Ecological Natural Resources Teaching Area and Coulter Airfield, respectively). ADH values were calculated using a base temperature of 10 °C and incorporated published development data for *Co. macellaria* (Boatright and Tomberlin 2010), the dominant summer blow fly species in the region.

Predicted ADH was used to determine when to remove carcasses from the field, based on the time required to reach the onset of third instar wandering behavior for *Co. macellaria* (∼4,500–5,000 ADH). To ensure consistency across replicates within each trial, all carcasses were removed simultaneously once the predicted ADH was reached. Throughout the entire trial, carcasses were left untouched to prevent disruption of larval masses or alterations to the microenvironment. The removal from the field based on developmental timing facilitated a standardized comparison of emergence patterns. During transit, pans were covered with breathable mesh secured by elastic bands to maintain airflow while preventing contamination or larval escape (Sawyer et al. 2022).

### Adult Fly Collection

Once in the lab, carcasses were placed in a walk-in environmental chamber set at ∼27 °C, 75% relative humidity (RH), and 12:12 L:D. Emergence was recorded at 1000 h daily for each carcass, allowing classification into first-emergence and last-emergence groups. When adults emerged, the pan was moved to a pop-up collection tent (81 cm × 81 cm × 136 cm) in an adjacent room (∼25 °C, ∼70% RH) where flies were released and collected within ∼3 min. Pans were then returned to the chamber. No new emergence over 72 h per each carcass replicate indicated the endpoint. At this point, the remaining carcass material was weighed, bagged, and frozen (−20 °C). Collected adult flies were also frozen and stored at −20 °C in labeled zipper bags for later species identification (Whitworth 2019) and emergence analysis. Adults from Sarcophagidae and Muscidae were occasionally produced from some carcasses; however, these taxa were excluded from analyses as the present study focused specifically on validating Calliphoridae development and model accuracy.

### Estimated-to-Actual TOP Ratio (%) Calculations

Since flies were collected at 1000 h daily for the entirety of the study, it was possible to calculate an estimated TOP based on published accumulated degree days (ADD) and accumulated degree hours (ADH) development data for *Co. macellaria* reared at a constant temperature of 28.3 °C (Boatright and Tomberlin 2010). Field and lab temperatures averaged 28.4 °C across both 2023 and 2024 seasons. Although hourly on-site temperatures were not recorded, weather data were retrieved from Weather Underground, using records from Easterwood Airport (CLL), the nearest station with a complete hourly dataset. This approach reflects standard practices in forensic casework, where regional airport data are commonly used (Amendt et al. 2007). However, future studies should incorporate data loggers to capture localized temperature variation, which is known to influence insect development and may enhance the accuracy of estimates. Once TOP was estimated utilizing weather data and the Boatright and Tomberlin (2010) development study, it was compared to the actual TOP and incorporated into an estimated-to-actual TOP ratio (%). Ratios over 100% indicate the estimated TOP based on the development study exceeded the actual TOP, where 100% values indicate the estimated TOP aligned with the actual TOP. Accuracy was defined as the mean number of carcasses in which estimated TOP spanned the actual TOP, and precision was defined as the standard deviation of these means converted to days or hours regarding ADD or ADH, respectively.


$$\mathrm{Estimated}-\mathrm{to}-\mathrm{actual}\;\mathrm{TOP}\;\mathrm{ratio}\;(\%)=\frac{\mathrm{estimated}\;\mathrm{TOP}}{\mathrm{actual}\;\mathrm{TOP}}\;\times \;100\%$$

Accuracy=number of carcasses with estimates that include actual TOP

Precision=SD converted to days (ADD) or hours (ADH)

### Statistics

Due to a lack of blow fly colonization on some carcass types, only large birds, and small, medium, and large mammal carcasses were analyzed. Descriptive statistics were determined for each level of the following fixed factors: carcass type (small mammal, medium mammal, large mammal, and large bird), thermal summation method (ADD vs. ADH), emergence timing (first-emergence sampling vs. last-emergence sampling), development study tissue type (porcine vs. equine), and coexistence status (*Co. macellaria* only vs. mixed [*Co. macellaria* and *Ch. rufifacies* present together]). Group means and standard deviations were calculated using rank-transformed data to align with the violated assumptions of Shapiro–Wilk’s normality and Levene’s homogeneity. Accuracy, precision, and overestimation were only a single data point (e.g., totality of carcasses), so no statistical analyses were conducted.

### Full Factorial Rank-Transformed ANOVA

A full factorial design was employed to assess significant effects of the following categorical factors: carcass type (large bird, small mammal, medium mammal, and large mammal), thermal summation method (ADD vs. ADH), timing (first-emergence sampling vs. last-emergence sampling), and development study tissue type (porcine vs. equine). A fifth factor, coexistence (*Co. macellaria* only vs. mixed), was included to capture the effect of coexistence on TOP ratios. Assumptions of homogeneity of variance were violated (Levene’s test, *P* < 0.05), and residuals showed minimal deviation from normality (Shapiro–Wilk *P* = 0.028); therefore, a ranked ANOVA was performed using rank-transformed TOP estimates as the dependent variable. The full factorial rank-transformed ANOVA included all main effects and interaction terms. Analyses were conducted in R v4.3.1 using base aov() functions, and data were reshaped via the tidyverse() function. Statistical outputs were reported with mean ± standard deviation (SD), with significance assessed at α = 0.05.

### Overestimation Calculations

To assess overestimation for the TOP—a clear error—we calculated the ratios of carcass types that overestimated TOP (see Table 1). Estimates were categorized by development study tissue type (porcine vs. equine), emergence timing (first or last *Co. macellaria* emergence), thermal summation method (ADD vs. ADH), and species coexistence (mixed vs. single). An estimate was considered an overestimation if the calculated TOP exceeded the known actual placement time. Overestimation ratios were tabulated for each condition.

**Table 1.** Percent overestimation of time of placement (TOP) across carcass tissue type, emergence timing, thermal summation method, and species coexistence

	Mixed—ADD (%)	Mixed—ADH (%)	Single—ADD (%)	Single—ADH (%)
**Equine—first-emergence**	26.6	0.07	27.8	0.0
**Equine—last-emergence**	0.0	0.0	0.0	0.0
**Porcine—first-emergence**	26.6	0.0	27.8	0.06
**Porcine—last-emergence**	0.0	0.0	0.0	0.0

Values reflect the percentage of carcass replicates whose estimated TOP exceeded the actual TOP.

## Results

### Estimated-to-Actual TOP Ratio (%)

**Fig. 1.**
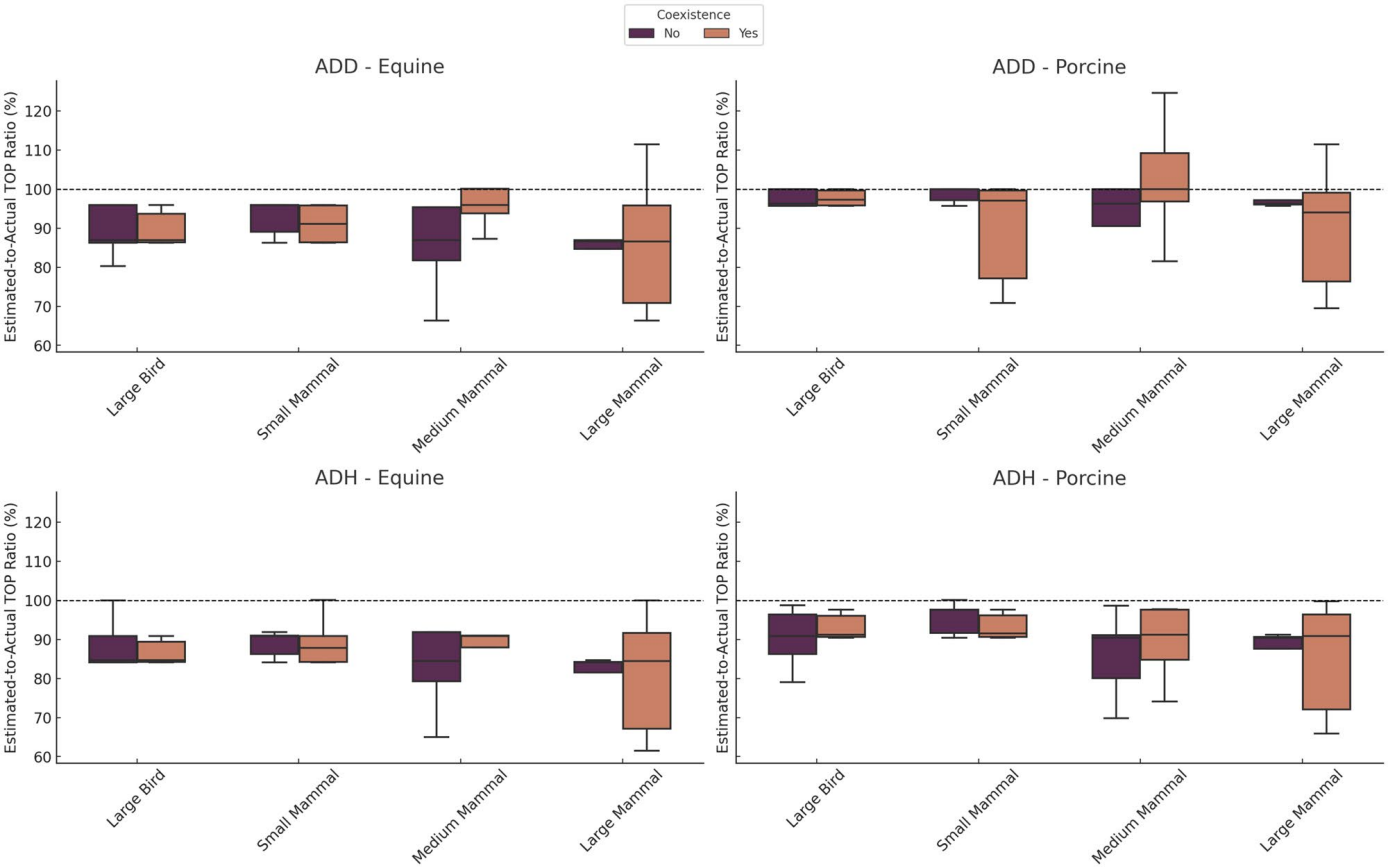
Boxplot panels display thermal summation method (ADD vs. ADH) and development study tissue type (equine vs. porcine), depicting estimated-to-actual TOP ratios (%) across carcass types. Colors indicate coexistence status: not mixed (No- *Co. macellaria* only) vs. mixed (Yes- *Co. macellaria* and *Ch. rufifacies*).

**Fig. 2.**
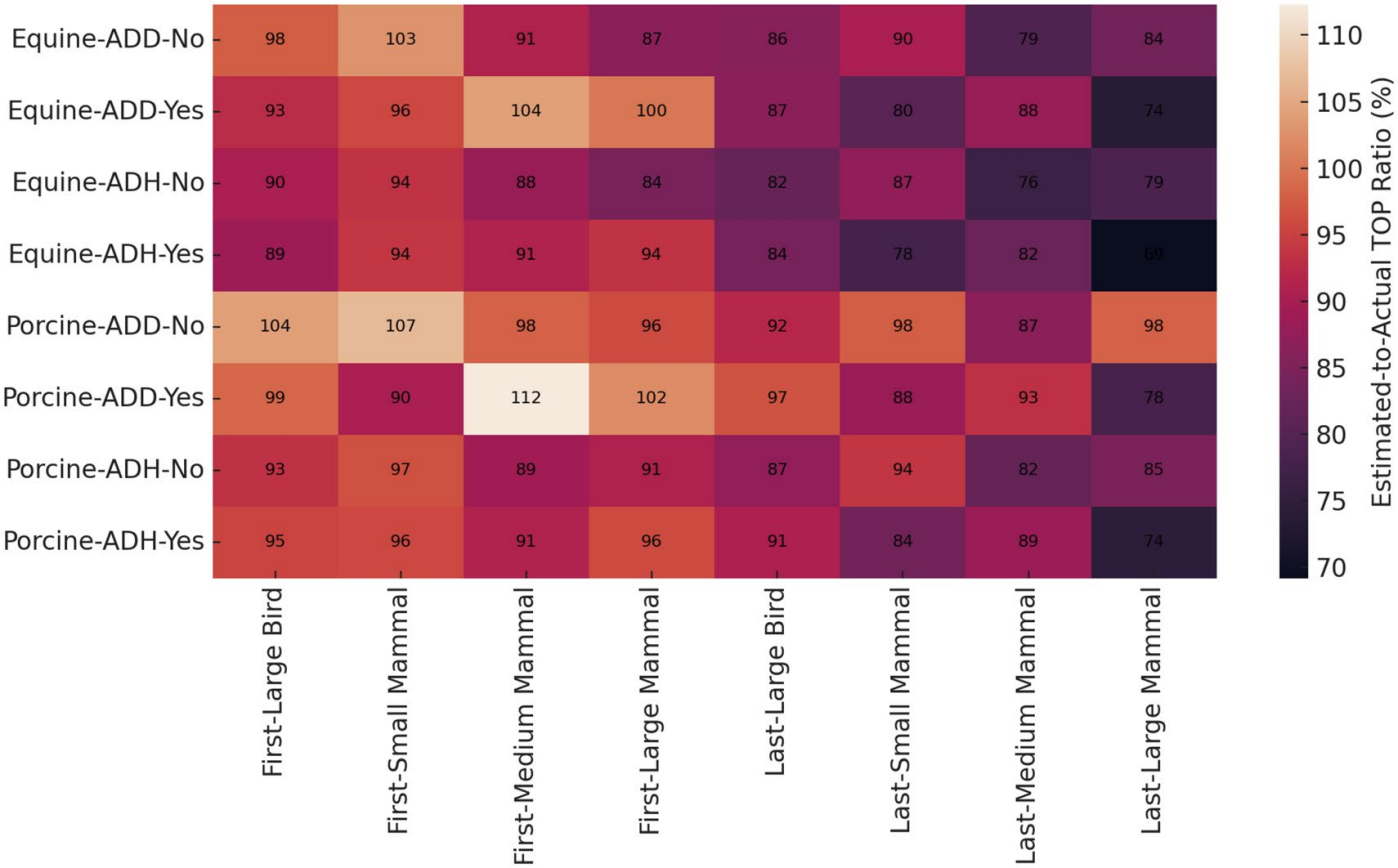
Heatmap depicting average estimated-to-actual TOP ratios (%) across carcass type, *Co. macellaria* emergence timing (i.e., first vs. last) (*x* axis) and factorial configuration (Development study tissue type, ADD vs. ADH, and species coexistence; *y* axis). Darker shaded cells indicate higher TOP ratios using either accumulated degree days (ADD) or hours (ADH) from equine or porcine development data under single-species (No—*Co*. *macellaria*-only) or mixed (Yes—*Co. macellaria* and *Ch. rufifacies*) colonization scenarios. Estimated-to-actual TOP ratio (%) values are depicted in each cell.

Means and standard deviations of TOP ratios were evaluated across carcass type (large bird, small mammal, medium mammal, and large mammal), thermal summation method (ADD vs. ADH), emergence timing (first vs. last), development study tissue type (porcine vs. equine), and species coexistence status (*Co. macellaria* only vs. mixed) (Figs 1 and 2). Among carcass types, small mammal carcasses produced highest ratios on average (93.8% ± 9.7%), followed by large birds (91.7% ± 8.5%) and medium mammals (90.1% ± 12.5%). Large mammal carcasses produced the lowest ratios (86.4% ± 13.3%), indicating greater variability in insect colonization dynamics as vertebrate biomass increases. Thermal summation method significantly influenced ratio values, with ADD-based ratios (93.9% ± 12.1%) outperforming those based on ADH (87.9% ± 9.2%). Timing of *Co. macellaria* emergence played a key factor: the first-emergence factor produced higher and less variable ratios (96.3% ± 8.9%) compared to last-emergence (85.4% ± 10.5%). Additionally, ratio means differed by the tissue origin used in the development dataset: porcine-derived values produced slightly higher ratios (93.4% ± 11.0%) than equine-derived values (88.3% ± 10.7%). Finally, species coexistence modestly impacted ratio means. Carcasses colonized solely by *Co. macellaria* yielded slightly higher ratios (91.8% ± 9.8%) than those co-colonized with *Ch. rufifacies* (89.6% ± 12.6%).

### Rank-Transformed Full-Factorial ANOVA

A full factorial aligned rank-transformed ANOVA was conducted to assess the effects of carcass type, thermal summation method (ADD vs. ADH), emergence timing (first vs. last), development study tissue type (porcine vs. equine), and species coexistence (mixed vs. *Co. macellaria* only) on the TOP ratios (%) based on *Co. macellaria* emergence data (Supplementary Table S1). Significant main effects were detected for carcass type (*F*_3_,_216_ = 9.12, *P* < 0.001), thermal summation method (*F*_1_,_216_ = 32.29, *P* < 0.001), emergence timing (*F*_1_,_216_ = 127.58, *P* < 0.001), and development study tissue type (*F*_1_,_216_ = 24.39, *P* < 0.001). Co-colonization with *Ch. rufifacies* (Mixed colonization status) had no significant main effect (*F*_1_,_216_ = 2.44, *P* = 0.120) on TOP ratios. Although variation in *Co. macellaria* emergence appeared much greater (up to 2×) when *Ch. rufifacies* was present (Fig. 1). For example, for ADD—equine/large mammal, the standard deviation is 5.3× greater under coexistence. The IQR is 11.45× larger, and the coefficient of variation (CV) is 5.2× greater, indicating substantial variability introduced by mixed-species conditions. Several interaction effects were also statistically significant. A significant carcass type × emergence timing interaction (*F*_3_,_216_ = 5.49, *P* = 0.0012) demonstrates that the influence of emergence timing on TOP ratio outcomes varied according to carcass type. A carcass type × species coexistence interaction (*F*_3_,_216_ = 6.88, *P* < 0.001) and a timing × species coexistence interaction (*F*_1_,_216_ = 7.76, *P* = 0.006) revealed context-dependent effects of mixed colonization on TOP ratios. A significant three-way interaction between carcass type, timing, and species coexistence (*F*_3_,_216_ = 8.11, *P* < 0.001) was also observed, indicating the impact of mixed-species colonization is not uniform but influenced by both carcass type and emergence timing. No other two-, three-, or four-way interactions involving tissue type, method, or mixed-species presence were statistically significant (*P* > 0.05).

### Post-Hoc Comparisons

Post-hoc pairwise comparisons using Tukey-adjusted estimated marginal means (emmeans) revealed several significant differences in TOP ratios across main effects and interactions. For carcass type, TOP ratios were significantly greater for small mammals compared to medium mammals (*P* = 0.005), and for large birds compared to large mammals (*P* = 0.020). Among thermal summation methods, ADD-based predictions produced significantly higher TOP ratios than ADH-based predictions (*P* < 0.001). Similarly, emergence timing had a strong effect, with first emergence predictions yielding significantly greater ratios than those based on last-emergence (*P* < 0.001). Development study tissue type also had a significant influence, with porcine-derived developmental data producing greater TOP ratios than equine-derived data (*P* < 0.001). Several interactions were significant. A notable carcass type × timing interaction showed that first-emergence estimates upon small mammals produced greater TOP ratios than last-emergence estimates from large mammals (*P* < 0.001). Likewise, the carcass type × mixed interaction indicated higher ratios in large birds without *Ch. rufifacies* coexistence compared to large mammals with coexistence (*P* = 0.002). Finally, a significant Timing × Mixed interaction demonstrated that first-emergence estimates without species coexistence produced greater TOP ratios than last-emergence estimates with coexistence (*P* < 0.001). Finally, a three-way interaction among Carcass Type, Timing, and Mixed showed that TOP ratios were greatest for small mammals at first emergence without *Ch. rufifacies*, compared to medium mammals at last-emergence with coexistence (*P* < 0.001).

### Accuracy, Precision, and Overestimation

High accuracy refers to TOP ratios at or above 90%, indicating that the estimated TOP was within 10% of the actual TOP. Forensically, this level of accuracy reflects stronger alignment between TOP estimates and actual TOP, supporting the reliability of laboratory-derived data within field conditions. Overall, estimates of TOP using ADD with development data for *Co. macellaria* reared on porcine tissue (Boatright and Tomberlin 2010) demonstrated higher accuracy (eg, 33% for large birds to 100% for small mammals) than those reared on equine tissue (eg, 0% for medium mammals to 42% for small mammals) (Table 1). For ADH, *Co. macellaria* development data for either tissue used in Boatright and Tomberlin (2010) performed similarly; however, it is important to note that accuracy was contingent on coexistence status (Table 1).

**Table 2.** Accuracy of time of placement (TOP) estimates based on emergence of *Co. macellaria* across carcass type, tissue source, thermal summation method (ADD vs. ADH), and species coexistence status

*Cochliomyia macellaria*	Accuracy = number of estimates per carcass type that reach the actual TOP				
Carcass type	Coexistence	ADD equine	ADD porcine	ADH equine	ADH porcine
**Large bird**	Single	1/3	1/3	0/3	0/3
**Large bird**	Mixed	2/7	5/7	1/7	0/7
**Small mammal**	Single	0/2	1/2	1/2	0/2
**Small mammal**	Mixed	3/7	7/7	2/7	1/7
**Medium mammal**	Single	0/4	2/4	0/4	0/4
**Medium mammal**	Mixed	2/5	5/5	0/5	0/5
**Large mammal**	Single	0/2	1/2	1/2	0/2
**Large mammal**	Mixed	2/5	3/5	2/5	0/5

Accuracy is reported as the number of estimates per treatment in which the predicted TOP overlapped with the actual TOP. Estimates were based on development data using equine or porcine tissue. “Mixed” indicates coexistence with *Ch. rufifacies*.

With regards to precision, ADD estimations of TOP ratio using development data for *Co. macellaria* reared on porcine tissue (Boatright and Tomberlin 2010) demonstrated greater precision (eg, 0.3 d for small mammals to 0.6 d for medium mammals) compared with those on equine tissue (eg, 0.4 d for small mammals to 2.8 d for large mammals) (Table 2). The opposite pattern is seen in ADH estimations of TOP using development data for *Co. macellaria* reared on equine tissue (Boatright and Tomberlin 2010), demonstrating greater precision (eg, 0.4 h for large mammals to 21.2 h for medium mammals) than those on porcine tissue (eg, 0.3 h for large birds to 38.3 h for medium mammals). Similarly, to results for accuracy, it is important to note that precision was contingent on coexistence status (Table 2).

**Table 3.** Precision of time of placement (TOP) estimates for *Co. macellaria* based on the standard deviation (SD) of estimated-to-actual TOP ratios

*Cochliomyia macellaria*		Precision = SD converted to days or hours			
Carcass	Coexistence	ADD equine (days)	ADD porcine (days)	ADH equine (hours)	ADH porcine (hours)
**Large bird**	Single	1.1	0.4	13.1	25.4
**Large bird**	Mixed	2.6	0.5	0.5	0.3
**Small mammal**	Single	2.0	0.4	12.2	13.7
**Small mammal**	Mixed	0.4	0.3	0.6	18.6
**Medium mammal**	Single	0.8	0.6	0.5	29.3
**Medium mammal**	Mixed	0.6	0.6	21.2	38.3
**Large mammal**	Single	2.8	0.5	0.4	0.3
**Large mammal**	Mixed	2.5	0.5	0.4	11.8

Precision is shown as SD converted to days (from ADD) or hours (from ADH), stratified by carcass type, species coexistence status (*Co. macellaria* only vs. mixed), and development tissue type (equine vs. porcine). Lower values reflect greater precision.

Overestimation of TOP occurred almost exclusively in first emergence TOP ratios using ADD thermal summation methods (Table 1). Specifically, equine-based ADD configurations overestimated TOP in 26.6% of mixed trials and 27.8% of single-species trials. Porcine-based ADD configurations similarly overestimated TOP in 26.6% and 27.8% of mixed- and single-species trials, respectively. Minimal overestimation was observed for ADH TOP estimations under any condition, and last-emergence estimates consistently aligned with or underestimated actual TOP. These results indicate the TOP estimates using ADD, when used with first emergence data, may introduce an upward bias in TOP estimates regardless of tissue type or species coexistence.

## Discussion

The use of TOP ratios (%) provided a quantitative framework for evaluating the degree of overestimation, accuracy, and precision of TOP estimates when applying laboratory-derived development data to *Co. macellaria* emergence under field conditions for different carcass types and sizes (Tables 1–3). Overall, estimates of TOP ratios were highly accurate across carcass types with or without *Ch. rufifacies*, supporting the applicability of published developmental datasets (eg, Boatright and Tomberlin 2010) to forensic scenarios when environmental conditions are comparable. As stated previously, accuracy was defined as the mean number of carcasses for which the estimated TOP spanned the actual TOP, while precision was expressed as the standard deviation of these means converted to days (ADD) or hours (ADH).

Among the factors tested, carcass type significantly influenced estimates of TOP ratios. TOP ratios from small mammal carcasses yielded the highest TOP ratio (93.8% ± 9.7%), while large mammal carcasses produced the lowest (86.4% ± 13.3%), likely reflecting ecological differences in decomposition dynamics (Gruner et al. 2017), thermal variability (Gbenonsi and Higley 2025), and surface area-to-volume ratios (Rivers et al. 2011). These findings underscore the necessity of considering carcass-specific factors when interpreting insect development patterns in forensic casework. Moreover, the increased variability observed in larger carcasses highlights the importance of incorporating best-practice recommendations from forensic guidelines (National Institute of Standards and Technology [NIST], National Institute of Justice [NIJ] 2017, Organization of Scientific Area Committees [OSAC] 2021), such as sampling from multiple larval masses to capture intra-carcass developmental heterogeneity.

Tissue type used in Boatright and Tomberlin (2010) also exhibited a significant main effect, with development data from porcine tissue producing higher TOP ratios (93.4% ± 11.0%) than equine-derived data (88.3% ± 10.7%). Although previous work reported negligible differences in *Co. macellaria* development across tissue types (Boatright and Tomberlin 2010), other species such as *Ch. rufifacies* demonstrate tissue-specific developmental plasticity (Flores et al. 2017). The discrepancy observed here (ie, development data based on tissue type had an impact) may be due to interactions between tissue composition (Bernhardt et al. 2018), microbial activity (Pechal et al. 2013), or larval density (Saunders and Bee 2013)—factors not fully captured in laboratory conditions.

Emergence timing had the most pronounced effect on estimates of TOP ratios. Those based on first-emergence were significantly (Supplementary Table S1) higher and less variable (96.3% ± 8.9%) than those for last-emergence (85.4% ± 10.5%). These data support the forensic standard of prioritizing samples of possible oldest immature insects when estimating TOC (Amendt et al. 2007), as they are more representative of initial colonization events. In contrast, last-emergence data may reflect developmental outliers or delayed colonization events, reducing predictive reliability. The discrepancy emphasizes the challenges of determining what to sample given insects collected may in fact not be the oldest.

Thermal summation method also impacted estimates of TOP ratios. ADD-based predictions of the estimates of TOP ratios outperformed ADH-based predictions (93.9% ± 12.1% vs. 87.9% ± 9.2%, respectively) (Supplementary Table S1), indicating ADD calculations may be more robust in response to/in the face of short-term thermal fluctuations, which can disproportionately impact ADH estimations. Furthermore, validation of development datasets under field conditions remains a critical step toward improving TOP estimation accuracy and precision (eg, TOC and minPMI). As demonstrated in the current study with ADD, the tissue type used in Boatright and Tomberlin (2010) impacted accuracy of TOP estimates with porcine—tissue, rather than equine—tissue, providing the greatest accuracy (Table 2) and precision (Table 3). The inverse was observed when applying ADH. With regards to overestimation of the TOP, regardless of tissue type (i.e., equine vs. porcine) and coexistence, ADH had minimal overestimation compared to ADD calculations for first-emergence (Table 1). No overestimations were generated when applying data to last-emergence, which is logically expected. Forensic practitioner guidelines suggest that ADH values are frequently rounded or reported to the nearest day rather than the hour, and the results of this study demonstrate that rounding to the day may reduce variability in ADH-based estimates. However, this practice may introduce over- or underestimation, particularly when daily temperature fluctuations are more extreme. In turn, the choice and reporting of the thermal summation model used should be transparent, and practitioners should consider the potential for error that is introduced when rounding.

In addition, when using the porcine development data from Boatright and Tomberlin (2010) to estimate TOP, coexistence with *Ch. rufifacies* impacted accuracy (Table 2), but not precision (Table 3). Furthermore, these results were only evident when applying ADD instead of ADH. This discrepancy could be due to the level of precision being applied in the calculations (eg, days vs hours). However, typically in casework, the application of ADH is not specific to the hour estimated for TOC, but rather generalized to the half day (eg, morning or afternoon). If this step were applied to the current estimates (Table 1), the discrepancy could decrease, regardless of whether using ADD or ADH.

Furthermore, while species coexistence (i.e., presence of *Ch. rufifacies*) was not a significant main effect for estimates of TOP ratios, it emerged as a key variable in several interaction terms, which is demonstrated when examining accuracy and precision as previously discussed. TOP ratios were highest, and less variable, when *Co. macellaria* occurred alone and when estimates were used first-emergence data and porcine-based ADD parameters. Interactions between *Co. macellaria* and *Ch. rufifacies*, a facultative predator (Brundage et al. 2014), as previously indicated, may accelerate *Co. macellaria* development through both consumptive and non-consumptive effects, such as altered behavior or exposure to predator excretions (Flores et al. 2017). The ecological realism of these findings is further supported by the observation that *Co. macellaria* co-occurred with *Ch. rufifacies* on over half of all carcasses. Thus, mixed-species colonization events should be considered the rule rather than the exception in forensic decomposition scenarios.

A significant three-way interaction among carcass type, emergence timing, and species coexistence further illustrates the context dependency of forensic applications. For example, estimates of TOP ratios for small mammal carcasses under single-species conditions and first-emergence sampling were significantly higher compared to large birds under mixed-species colonization and last-emergence sampling. Collectively, these results indicate TOP estimates are most accurate when derived from first-emergence ADD values using porcine tissue development data, particularly for smaller mammal or larger bird carcasses under single-species colonization conditions. This finding indicates emergence timing, thermal summation method (ADD vs. ADH), and development study tissue type are the primary drivers of TOP ratio accuracy. However, their influence is further moderated by carcass composition and the presence of mixed-species colonization. Notably, increased vertebrate biomass appears to introduce greater variability in colonization dynamics and larval development rates, potentially complicating TOP estimation under more complex ecological conditions. Despite the strength of these findings, this study was limited to a single regional population of *Co. macellaria* in Central TX, United States. Given prior evidence of geographic variation in blow fly development (Gallagher et al. 2010, Owings et al. 2014), extrapolation to other climates or populations should be approached cautiously. Nutritional content and tissue composition (eg, fat, protein, moisture) may differ between whole-carcass types and could influence blow fly development (Pechal et al. 2013, Flores et al. 2017, Bernhardt et al. 2018). While the goal was to evaluate the generalizability of published developmental timelines, tissue quality remains a potential confounding factor requiring further investigation. This study simulated first and last-emergence as proxies for field sampling; however, forensic practitioners typically collect subsamples, not entire larval masses. Development of subsampling guidelines for application (e.g., casework and validation studies) and integration with rearing protocols is a critical next step for improving forensic application reliability. Future validation efforts should be conducted with human remains. Such efforts should be expanded to other regions globally and should be conducted with vertebrate remains (eg, fully intact human remains) that have a known time and cause of death (i.e., minPMI) to potentially increase the ecological and legal relevance of estimates generated. These findings underscore the importance of ecological context in applying developmental data—not only for forensic casework, but also for vector ecology. Blow flies can act as mechanical vectors of pathogens, and understanding their development under natural conditions enhances our ability to assess their role in pathogen transmission within decomposing systems and other public health contexts.

## Supplementary Material

tjaf167_Supplementary_Data
