# Supplementary material for: Validating the use of a local developmental dataset for Cochliomyia macellaria (Diptera: Calliphoridae) to estimate the time of placement of various carrion types and sizes in Texas, United States, during summer
Source: J Med Entomol. 2025 Nov 14;63(1):tjaf167. doi: 10.1093/jme/tjaf167 (PMC13329186; doi:10.1093/jme/tjaf167)
Supplement: tjaf167_Supplementary_Data [file tjaf167_supplementary_data.pdf]

Supplemental Table 1. Output table for rank-transformed full factorial ANOVA including main effects for carcass type (LB, SM, MM, LM), method (ADD VS. ADH), timing (first-emergence vs. last-emergence), coexistence (*Co. macellaria* only vs. mixed), and development study tissue type (equine vs. porcine), as well as all their interaction effects and the residuals.

**Effect**	**df**	**F value**	**P (>F)**
CarcassType	3	9.117	**0.000**
Method	1	32.289	**0.000**
Timing	1	127.580	**0.000**
Mixed	1	2.435	0.120
TissueType	1	24.389	**0.000**
CarcassType × Method	3	0.308	0.820
CarcassType × Timing	3	5.485	**0.001**
Method × Timing	1	1.872	0.173
CarcassType × Mixed	3	6.876	**0.000**
Method × Mixed	1	0.001	0.970
Timing × Mixed	1	7.762	**0.006**
CarcassType × TissueType	3	0.050	0.985
Method × TissueType	1	0.732	0.393
Timing × TissueType	1	1.490	0.224
Mixed × TissueType	1	0.261	0.610
CarcassType × Method × Timing	3	0.393	0.758
CarcassType × Method × Mixed	3	0.986	0.400
CarcassType × Timing × Mixed	3	8.108	**0.000**
Method × Timing × Mixed	1	0.005	0.944
CarcassType × Method × TissueType	3	0.092	0.964
CarcassType × Timing × TissueType	3	0.123	0.947
Method × Timing × TissueType	1	0.009	0.923
CarcassType × Mixed × TissueType	3	0.663	0.576
Method × Mixed × TissueType	1	0.168	0.683
Timing × Mixed × TissueType	1	0.008	0.928
CarcassType × Method × Timing × Mixed	3	0.998	0.395
CarcassType × Method × Timing × TissueType	3	0.232	0.874
CarcassType × Method × Mixed × TissueType	3	0.102	0.959
CarcassType × Timing × Mixed × TissueType	3	0.078	0.972
Method × Timing × Mixed × TissueType	1	0.000	0.987
CarcassType × Method × Timing × Mixed × TissueType	3	0.179	0.911
Residuals	216		
